# *Citrobacter* Species Increase Energy Harvest by Modulating Intestinal Microbiota in Fish: Nondominant Species Play Important Functions

**DOI:** 10.1128/mSystems.00303-20

**Published:** 2020-06-16

**Authors:** Mei-Ling Zhang, Miao Li, Yi Sheng, Fang Tan, Liqiao Chen, Isaac Cann, Zhen-Yu Du

**Affiliations:** a School of Life Sciences, East China Normal University, Shanghai, China; b Department of Animal Sciences, University of Illinois at Urbana–Champaign, Urbana, Illinois, USA; c Department of Microbiology, University of Illinois at Urbana–Champaign, Urbana, Illinois, USA; d Carl R. Woese Institute for Genomic Biology, University of Illinois at Urbana-Champaign, Urbana, Illinois, USA; e Division of Nutritional Sciences, University of Illinois at Urbana-Champaign, Urbana, Illinois, USA; Duke University School of Medicine

**Keywords:** energy harvest, gut microbiota, high-fat diet, fish, intestinal permeability

## Abstract

An efficient energy harvesting mechanism is likely critical for animals in their natural environment. Intestinal microbiota enriched by a high-fat diet aid in lipid accumulation, a strategy likely evolved for energy harvest in mammals. However, whether this strategy is conserved among vertebrate organisms remains unclear. A bacterial strain (S1), enriched on soybean oil rich medium, was isolated from the gut of Nile tilapia and demonstrated to be a member of the *Citrobacter* genus. Although a high-fat diet increased the number of *Citrobacter* spp., these bacteria were not abundant in the intestine by high-throughput sequencing. Addition of bacterium S1 to a high-fat diet modulated intestinal microbial composition and increased high-fat diet-induced lipid accumulation in mesenteric adipose tissue, accompanied by (i) increased triglyceride absorption efficiency and triglyceride reesterification and (ii) increased intestinal permeability. Collectively, our results provide evidence that specific intestinal bacteria aid the host in harvesting more energy from a high-fat diet in fish. Furthermore, the results from the present study also suggest that nondominant bacteria in the gut may play an important role in regulating host metabolism.

**IMPORTANCE** This study shows that the ability of gut microbiota members to enhance host energy harvest from a high-fat diet is a conserved feature of host-microbe interactions in fish, as in mammals. It also underscores that gut microbiota members are able to significantly impact host biology even when at low abundance.

## Abstract (precis)

This study shows that the ability of gut microbiota members to enhance host energy harvest from a high-fat diet is a conserved feature of host-microbe interactions in fish, as in mammals. It also underscores that gut microbiota members are able to significantly impact host biology even when at low abundance.

## INTRODUCTION

Throughout animal evolution, energy conservation mechanisms have been vitally important for survival, because food intake was irregular and scarce (1). Accumulated fat can serve as an energy reserve, and this will increase the survival rate of individuals during food shortage (2). From this point of view, harvesting more energy from diet is an adaption strategy for organisms to survive in their natural habitats (3).

Intestinal microbiota coevolves with the human host and complements the coding potential of the human genome with 500-fold more genes (4). Thousands of bacterial phylotypes are deeply involved in a series of host metabolism steps, and intestinal microbiota-host cross talk is crucial for energy harvest (5, 6). It has been found that germfree (GF) rats obtain less energy from polysaccharide-rich diets, and germfree mice accumulate less adiposity, even when they have an increased intake of food compared with conventionalized mice (5, 7). Compared to specific-pathogen-free (SPF) mice, GF mice are resistant to diet-induced obesity, which may be due to impaired lipid digestion and absorption, while GF mice conventionalized with high-fat-diet (HFD)-induced jejunal microbiota exhibit increased lipid absorption, suggesting that the intestinal microbiota enriched by high-fat diet contributes to lipid accumulation in mice (8). Several bacteria have been found to contribute to lipid accumulation by different mechanisms in mammals (9, 10). As examples, Clostridium bifermentans and Lactobacillus rhamnosus GG could upregulate the expression level of *Dgat2* to facilitate lipid absorption (8). It has been reported that lipopolysaccharide (LPS) secreted by a bacterium may cause low-grade inflammation and induce lipid accumulation in mice (9). Furthermore, enhanced transport efficiency of nutrients, including glucose and fatty acids, was observed when germfree mice were colonized with a simplified human intestinal microbiota together with Clostridium ramosum (11). However, whether the role of intestinal bacteria in assisting hosts to harvest energy is conserved among taxa is still unknown.

Evolutionarily, fish are more primitive than mammals, but like mammals, fish accumulate extra lipid when fed a high-fat diet (12), and diet components could influence the intestinal microbiota in fish (13). It is of significance, however, that fish harbor a *Proteobacteria*-dominated microbiota, which is different from the dominant microbiota in humans or mice (14). One work based on zebrafish indicated that the presence of diet could enrich the proportion of *Firmicutes* compared with that in a starvation group, and furthermore, diet-enriched *Firmicutes* and their products could increase lipid droplet number or size in intestinal epithelial cells. These observations suggested that the intestinal microbial community can act as a target for controlling dietary fat absorption in fish (6). Therefore, we hypothesized that even in fish, which have an intestinal microbiota composition largely different from that of mammals, intestinal bacteria can regulate lipid metabolism and contribute to energy harvest when dietary energy is abundant.

Nile tilapia (Oreochromis niloticus) is an important aquaculture species, and it is a common fish model for nutrition and metabolism studies (12, 15). In the present study, a high-fat diet containing soybean oil, was used to feed Nile tilapia to induce lipid accumulation (12), a function-based approach was used to isolate a bacterium that may help calorie harvesting in fish, and the possible mechanism was identified. By investigating intestinal bacterial diversity in fish, we observed that bacterium S1, a nondominant bacterium in the fish gut, increases fish lipid accumulation and modulates the intestinal microbial community. Understanding the function of nondominant bacteria in energy acquisition will expand our understanding of the physiological roles of intestinal microbiota; nondominant organisms may play critical roles in this important process.

## RESULTS

### Bacterium S1 is isolated by using soybean oil as the main carbon source.

**FIG 1:**
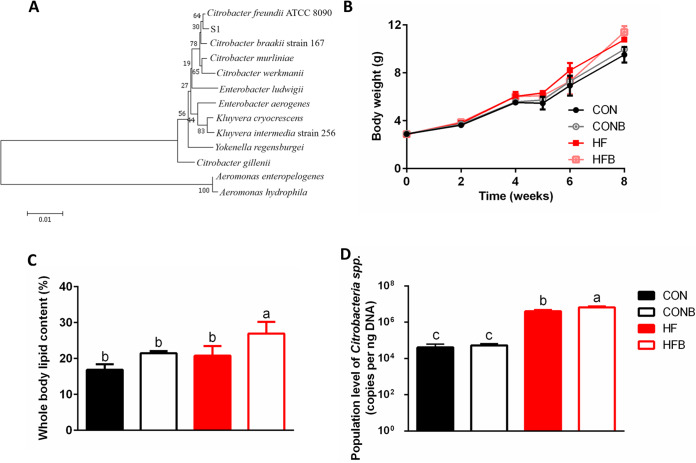
Isolating a bacterial strain *in vitro* and detecting its function *in vivo*. (A) Phylogenetic tree of the isolated bacterium. Distance was calculated based on the neighbor-joining criterion, and the bootstrap confidence values were 100 replicates. (B) Body weight during the experiment (*n* = 16). CON or CONB, fish were fed with basal diet without or with the isolated bacterium; HF or HFB, fish were fed with high-fat diet without or with the isolated bacterium. (C) Whole body lipid content (*n* = 3). (D) Quantification of *Citrobacter* spp. in four groups (*n* = 5). Data are expressed as mean values ± SEM. The different lowercase letters above each bar represent significant difference (*P < *0.05).

In order to isolate bacteria that are more prone to grow in a soybean oil-rich environment, soybean oil was selected as the main carbon source for culture media. After amplified rRNA gene restriction analysis (ARDRA), a bacterium which was more abundant *in vitro* was isolated and used for further research (designated S1). The 16S rRNA full-length gene sequence showed that the nearest neighbor of the isolated bacterium is Citrobacter freundii (Fig. 1A).

### The intestinal colonization of bacterium S1 and its effects on growth and body lipid content of fish.

To detect whether bacterium S1 could reach and proliferate in fish gut, one colony with rifampin resistance was selected and added to the diet of fish in the first feeding trial. Fecal material was plated on media containing rifampin to determine whether the rifampin-resistant strain can colonize the gut of Tilapia. Bacterium S1 could be detected in the gut of the fish fed with the diet supplemented with bacteria S1 from the 4th week after bacterium addition (Table 1).

**TABLE 1:** Determination of bacterial colonization efficiency

Group	CFU/g of feces at^*b*^:				
	4th wk	5th wk	6th wk	7th wk	8th wk
Without bacterium addition	ND	ND	ND	ND	ND
With bacterium addition^*a*^	1.88 × 10^5^	1.90 × 10^5^	1.48 × 10^5^	4.24 × 10^5^	6.20 × 10^5^

a A total of 10^9^ CFU of bacterium S1 were added per gram of diet.

b The intestinal content was cultured on a plate containing 700 μg ml^−1^ of rifampin.

With the aim of studying the influence of bacterium S1 on fish metabolism *in vivo*, bacterium S1 was added to the diet of the control or high-fat-diet group at a concentration of 10^9^ CFU g^−1^ of diet, and the growth characteristics of fish were examined. After 8 weeks of diet intervention, we found that supplementation with the bacterium did not significantly increase the body weight (Fig. 1B). However, addition of the bacterium S1in the high-fat diet increased the body lipid content significantly compared to those in other groups (Fig. 1C).

### Influence of high-fat diet or addition of bacterium S1 on the intestinal microbiota of fish.

To determine the population level of *Citrobacter* spp. in fish gut, genus-specific primers were used to quantify the abundance of *Citrobacter* spp. in the four groups (CON [fed with basal diet], CONB [fed with basal diet with 10^9^ CFU of S1 g^−1^ of diet added], HF [fed with high-fat diet], and HFB [fed with high-fat diet] with 10^9^ CFU of S1 g^−1^ of diet added). The results revealed that the population level of *Citrobacter* spp. increased significantly in high-fat diet group compared with the control group, suggesting that the high-fat diet favored the growth of *Citrobacter* spp. *in vivo* (Fig. 1D). Furthermore, addition of bacterium S1 significantly increased the abundance of *Citrobacter* spp. in the HFB group compared with that in the HF group (Fig. 1D). Two-way analysis of variance (ANOVA) also suggested that interaction of high-fat diet and the addition of bacterium S1 was correlated with the increased abundance of *Citrobacter* spp. (see Table S5 in the supplemental material).

**FIG 2:**
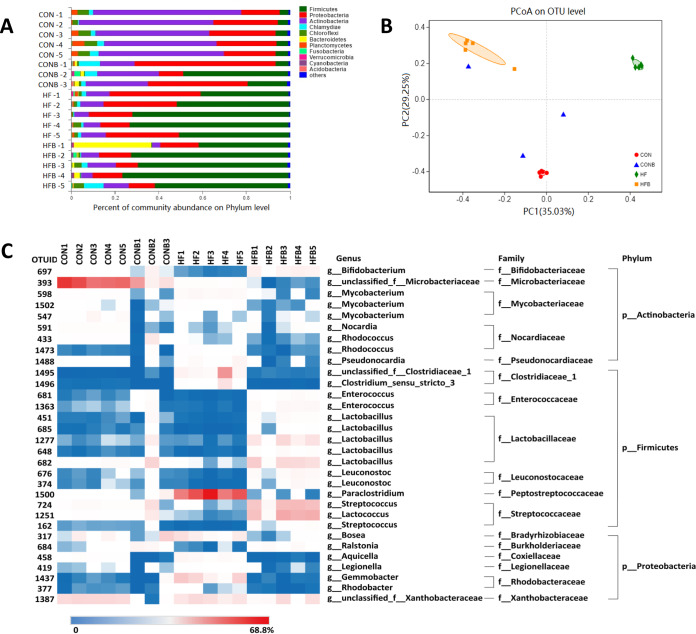
High-fat diet or addition of isolated bacterium induced significant changes in microbiota composition. (A) Community abundance of each group at the phylum level (*n* = 3 to 5). (B) OTU-based PCoA plot of samples from four groups. (C) Heat map analysis of 31 OTUs. The color bar of each OTU in each treatment is shown. The taxonomy of the OTUs (genus, family, and phylum) is depicted on the right. Differences were detected using Kruskal-Wallis in R package.

In order to detect whether *Citrobacter* spp. was dominant in fish gut, the V3-V4 region of the 16S rRNA gene was sequenced from genomic DNA extracted from five individuals randomly selected from each treatment. Two samples did not pass the sequencing quality check, so only three samples were involved in the CONB group. We did not find *Citrobacter*-related sequences in all sequencing data due to the low abundance of *Citrobacter* spp. compared with the whole intestinal microbial community. Compared with the whole intestinal microbial community, however, all samples did test positive in genus-specific quantitative PCR (qPCR). Instead, we noticed that the proportion of Firmicutes increased in high-fat or bacterial supplementation groups compared with the control group (Fig. 2A). The dominant phyla in the CON group were *Actinobacteria* (58.07%), *Proteobacteria* (25.71%), and *Firmicutes* (5.11%). In the CONB group, the most dominant phyla were *Proteobacteria* (40.30%), *Actinobacteria* (24.23%), and *Firmicutes* (23.98%). *Firmicutes* (58.15%), *Proteobacteria* (28.82%), and *Actinobacteria* (8.16%) were dominant in the HF group, and in the HFB group, *Firmicutes* (62.64%), *Proteobacteria* (13.36%), and *Actinobacteria* (8.18%) were more abundant. The relative abundances of *Firmicutes* were significantly increased in the HF and HFB groups compared with those in the control group.

The operational taxonomic unit (OTU)-based principal-coordinate analysis (PCoA) plot revealed that the individuals from the CON, HF, and HFB groups differed from one another (Fig. 2B). Compared with those in the control, the abundance-based coverage estimator (ACE) and Chao1 decreased significantly in the CONB group, while the HFB group showed higher Shannon index, ACE, and Chao1 than those of the HF group and lower Simpson index (*P* < 0.05) (Table S4). Two-way ANOVA suggested that lipid concentration in the diet may account for the change of Shannon and Simpson indexes, while interaction of the high-fat diet and the addition of bacterium S1 was correlated with ACE and Chao1 (Table S5).

Compared to those in the control group, the high-fat diet decreased the proportions of *Bifidobacterium*, *Nocardia*, *Microbacteriaceae*, *Enterococcus*, *Lactobacillus*, *Lactococcus*, *Leuconostoc*, *Streptococcus*, and *Ralstonia* (Fig. 2C). And among these genera, we found that some were induced by the addition of bacterium S1 in the high-fat-diet group, including *Bifidobacterium*, *Enterococcus*, *Lactobacillus*, *Streptococcus*, *Leuconostoc*, *Lactococcus*, and *Ralstonia*. The high-fat diet increased the abundances of *Paraclostridium*, *Gemmobacter*, *Rhodobacter*, *Clostridium*, and *Aquicella* significantly, while addition of bacterium S1 decreased the abundances of these genera. We also noticed that alteration of some OTUs, including OTU393 (*Microbacteriaceae*) and OTU433 (*Rhodococcus*), showed similar trends in response to high-fat diet or addition of bacterium S1. These results suggested that either high-fat diet or addition of bacterium S1 influenced the intestinal microbial composition, although bacterium S1 was not a dominant member of the fish gut microbial community.

### High-fat diet combined with bacterium S1 increased lipid accumulation in mesenteric adipose tissues.

**FIG 3:**
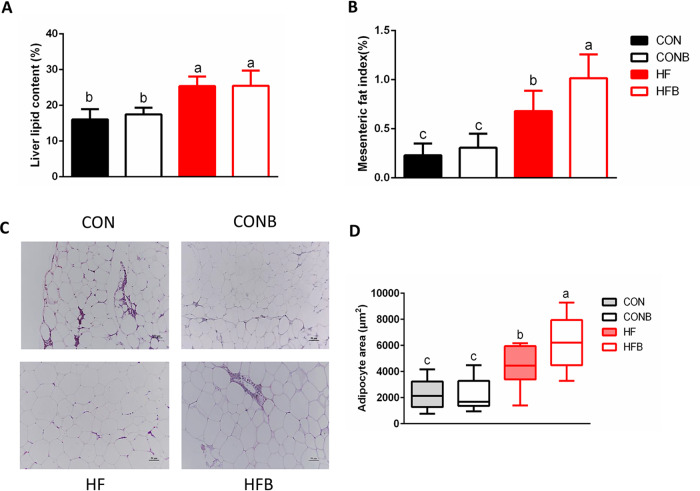
Lipid accumulation in the four experimental groups. (A) Liver lipid content (*n* = 6). (B) Mesenteric fat index (*n* = 6). (C) Hematoxylin and eosin (H&E) staining of mesenteric adipose tissue. Scale bars, 50 μm. (D) Mean adipocyte area, calculated by using ImageJ (3 slides per group). Bars headed by different letters were significantly different (*P < *0.05).

The liver and mesenteric adipose tissues are the main sites for lipid storage. Therefore, the lipid contents of these two tissues were determined in the four groups. The results indicated that the high-fat diet significantly increase the hepatic lipid content, but addition of bacterium S1 did not further lead to significant differences in the hepatic lipid content compared to that in the HF group (Fig. 3A, Fig. S1, and Table S5). The high-fat-diet group exhibited a higher mesenteric fat index than the control group (*P* < 0.05), and interestingly, we also found that addition of the bacterium S1 to the HFB group exacerbated the lipid accumulation in mesenteric adipose tissue compared with that in the HF group (*P* < 0.05) (Fig. 3B). The calculation of the size of adipocytes based on histological images also showed larger adipocytes in the HFB group than in the HF group (*P* < 0.05) (Fig. 3C and D). Two-way ANOVA also verified the influence of the interaction between the high-fat diet and the addition of bacterium S1 on the adipocyte area in mesenteric tissue (Table S5).

10.1128/mSystems.00303-20.7 FIG S1 Addition of the isolated bacterium yielded no significant difference on liver lipid content. H&E staining of liver tissue (scale bars, 50 μm). Download FIG S1, TIF file, 1.9 MB. Copyright © 2020 Zhang et al. 2020 Zhang et al. This content is distributed under the terms of the Creative Commons Attribution 4.0 International license.

### High-fat diet supplemented with the bacterium S1 group showed higher triglyceride absorption in the intestine.

**FIG 4:**
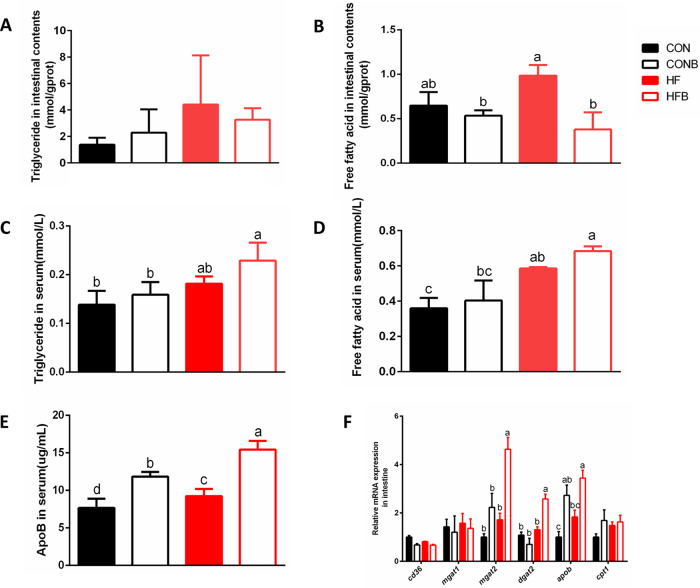
Triglyceride absorption and reesterification in the four experimental groups (*n* = 6). (A and B) Contents of triglycerides (A) and free fatty acids (B) in intestine. (C and D) Contents of triglycerides (C) and free fatty acids (D) in serum. (E) ApoB concentration in serum. (F) Expression levels of genes related to lipid metabolism. Mean values of six individuals ± SEM are shown. The different lowercase letters above each bar represent significant difference (*P < *0.05).

The above-described results suggested that the high-fat diet supplemented with the bacterium S1 increased the lipid accumulation. To verify this hypothesis, soybean oil was administered via oral gavage to fish to trace the absorption of triglycerides. The concentrations of triglycerides and free fatty acids in the intestinal content and serum were measured after 90 min of oral gavage. The results did not show significant differences in triglyceride in the intestinal contents among groups (Fig. 4A); however, addition of bacterium S1 decreased the concentration of fatty acids in the intestinal contents in the high-fat-diet treatments (*P* < 0.05) (Fig. 4B). The interaction between the high-fat diet and bacterium S1 was found to affect the concentration of fatty acids in the intestinal contents (Table S5). The concentrations of triglycerides and free fatty acids in the serum were higher in the HFB group than in the HF group, but no significant difference was found (Fig. 4C and D), and lipid concentration in the diet may account for these changes based on two-way ANOVA (Table S5). Considering that ApoB is crucial for the formation and secretion of intestinal chylomicron particles, the concentration of ApoB in the serum was also measured, and as expected, addition of bacterium S1 increased the concentration of ApoB in serum in both the control and high-fat-diet groups (*P* < 0.05) (Fig. 4E).

The expression levels of genes related to lipid metabolism were also determined in the foregut. The genes related to fatty acid uptake (*cd36*) and beta-oxidation (*cpt1*) did not show significant differences. Addition of bacterium S1 influenced the expression level of *mgat2* and *apob* (Fig. 4F). The correlative interaction of the high-fat diet and bacterium S1 was observed for the expression of *dgat2* (Table S5).

### Supplementation of high-fat diet with bacterium S1 increased intestinal permeability.

**FIG 5:**
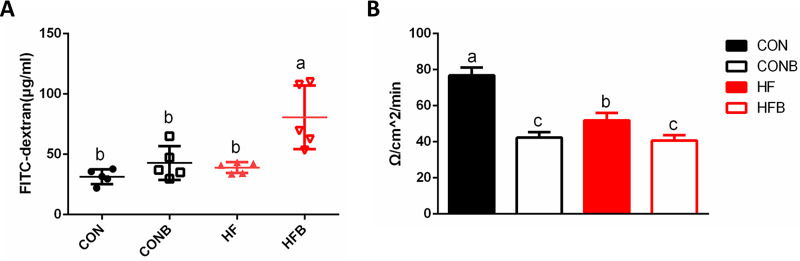
Intestine permeability in the four experimental groups (*n* = 6). (A) FITC-dextran concentration collected from the serum after oral gavage. (B) Ussing chamber recording of foregut from fish. Mean values ± SEM are shown. Different letters indicate significant difference (*P < *0.05).

Because intestinal permeability is linked to fat absorption (16), the influence of bacterium S1 on the intestinal permeability was also examined *in vivo* and *in vitro*. Enhanced influx of luminal molecules was observed in the HFB group compared with those in other groups (*P* < 0.05) when fish were administered fluorescein isothiocyanate (FITC)-conjugated dextran (molecular weight [MW], 4 kDa), but no significant difference was found between the CON and CONB groups (Fig. 5A). Decreased electrical resistance was observed in the high-fat-diet and bacterium-supplemented groups (Fig. 5B). Two-way ANOVA indicated that the interaction between the high-fat diet and the addition of bacterium S1 was related to intestinal permeability (Table S5).

## DISCUSSION

Diet-induced obesity (DIO) was characterized by flourishing of an uncultured clade within the phylum *Firmicutes*, and transplantation of the microbiota from mice with DIO to lean germfree recipients caused a significantly greater increase in adiposity than transplants from lean donors, suggesting intestinal microbiota alteration-linked diet-induced obesity (17). However, the relationship between the ratio of the *Firmicutes* phylum to the *Bacteroidetes* phylum and obesity remained controversial in the following studies (18). It should be noted that the ratio of the *Firmicutes* to the *Bacteroidetes* is reproducibly increased with high-fat-diet feeding in mice (19). Whether the response of intestinal microbiota to a high-fat diet is universal among hosts is unknown. Intestinal microbiota composition in fish is very different from those in mammals, with *Proteobacteria* being dominant in Nile tilapia, rather than *Firmicutes* and *Bacteroidetes*, reported as dominant in many mammals (20). In the present study, the high-fat diet increased the abundance of *Firmicutes*; thus, it appears that enrichment of *Firmicutes* in response to a high-fat diet is a general strategy in both mammals and fish. We also noticed that changes of different members in this phylum in response to the high-fat diet also differed; for example, *Paraclostridium* increased in the high-fat-diet group, but other members in *Firmicutes*, including *Enterococcus*, *Lactobacillus*, *Leuconostoc*, and *Streptococcus*, decreased in the high-fat-diet group but increased with the addition of bacterium S1 in the high-fat-diet group. Considering the possible function of these bacteria, including promoting fat storage or facilitating lipid absorption, the enriched members likely help the host to harvest or store more energy from the high-fat diets (8, 10, 21). It should be noted that a weakness of this present study is that the sample number for microbiota sequencing was relatively low. Although the microbiota composition of each individual sample appeared to be similar within each group, power calculations and adequate samples should be included in future studies to help control for variation between individuals and tanks.

The mechanism by which intestinal bacteria increased lipid accumulation has been attracting attention. As an example, an opportunistic pathogen isolated from an obese human was shown to elicit severe lipid accumulation in germfree mice. The possible reason has been ascribed to the LPS secreted by the bacterium and its likelihood to cause low-grade inflammation (9). Furthermore, Clostridium ramosum upregulated small intestinal glucose and fat transporters to induced obesity in gnotobiotic mice (11) and Lactobacillus paracasei promoted fat storage in enterocytes, although Escherichia coli enhanced lipid catabolism and reduced chylomicron circulating levels under homeostatic conditions (21). Inflammation markers and LPS binding protein content in serum were detected in the present study. However, no significant increase of the inflammatory markers was found in the HFB group compared with the levels in the HF group (Fig. S2), suggesting that inflammations were not the main drivers for lipid accumulation in the present study. To rule out the effect of addition of bacterium S1 on digestion efficiency, the lipase enzyme activity in the foregut was also measured; no significant difference was found between the groups with and without S1 (Fig. S3). It is known that Dgat2 is a critical enzyme for triglyceride synthesis and storage, and *Clostridiaceae* could upregulate the expression level of *Dgat2* to facilitate lipid absorption (8). In the present study, the interaction between the high-fat diet and bacterium correlated with the expression of *dgat2*; furthermore, a series of physiological steps involved in chylomicron secretion were also examined. From the results it could be concluded that administering bacterium S1 in the high-fat diet influenced the intestinal microbiota, which increased the efficiency of triglyceride absorption and transportation as the likely mechanism for lipid accumulation in the current study.

10.1128/mSystems.00303-20.8FIG S2Addition of the isolated bacterium (S1) did not induce significant inflammation. (A) Expression level of genes related to intestinal inflammatory factors. (B) Concentration of LPS binding protein in serum. Mean values of six individuals ± standard errors of the means (SEM) are shown. Download FIG S2, TIF file, 0.3 MB.Copyright © 2020 Zhang et al.2020Zhang et al.This content is distributed under the terms of the Creative Commons Attribution 4.0 International license.

10.1128/mSystems.00303-20.9FIG S3No significant difference was observed in lipase activity in four groups. Mean values of six individuals ± standard errors of the means (SEM) were shown. Download FIG S3, TIF file, 0.5 MB.Copyright © 2020 Zhang et al.2020Zhang et al.This content is distributed under the terms of the Creative Commons Attribution 4.0 International license.

Intestinal barrier dysfunction is also an important feature in obesity and metabolic syndrome (22). It has been found that rats given antibiotics had reduced intestinal permeability, and interestingly, these changes were associated with an unexpected decrease in lipid absorption. The likely reason ascribed to these observations was reduced ApoB secretion (16). Increased Bilophila wadsworthia augmented the impact of high-fat-diet-induced gut barrier alterations (10), suggesting that the intestinal integrity was influenced with the addition of this bacterium and therefore may be linked to increased lipid absorption. Increased intestinal permeability was also found in the HFB group in the current study. Since there was no obvious inflammation found in the intestine in the present study (Fig. S2), the change in intestinal permeability with the high-fat diet may also be an adaption toward environmental factors, similar to the alteration induced by intestinal microbiota.

Sequence-based and function-based strategies have often been used to identify bacteria that play important roles in host metabolism. A sequence-based method could facilitate the monitoring of the abundance of bacteria (9), but it is not suitable for nondominant members. A function-based method offers a more promising approach for the discovery of new bacterial candidates that may be nondominant in a community (23) but play significant roles in the community. This is illustrated in the present study, in which a function-based method was employed to isolate a bacterium affiliated with *Citrobacter*.

In earlier studies, Citrobacter freundii could be easily cultured with lipid emulsion (24), and an increase in abundance of this genus was observed in the high-fat-diet group. A subsequent study also showed that *B. wadsworthia*, enriched by a high-fat diet in mice, also induced lipid accumulation in the liver, although administering this bacterium in the study did not lead to major changes in microbiota composition (10). Our results, reported here, show that addition of bacterium S1 increased lipid accumulation in mesenteric adipose tissue; however, our observations differ in important aspects, since bacterium S1 is not a dominant member in the intestinal microbiota and yet it influenced the intestinal microbiota to modulate host metabolism. These results suggest that intestinal microbiota is coinvolved with the host for energy harvest and, importantly, nondominant bacterial members should not be ignored when we assess the function of intestinal microbiota. It should be noted that the abundances of *Citrobacter* spp. were similar between CON and CONB. One possible reason is that bacterium S1 may not thrive in fish gut when fish are fed with the control diet, and the other possible reason is that the primers used for quantification are not powerful enough to show the change of the strain. Therefore, in future studies, more powerful techniques, such as tagging the bacteria with metabolic probes (25), should be conducted to detect the colonization status of the intestinal bacteria, especially for nondominant ones.

The study reported here thus demonstrates that alteration of intestinal bacteria observed in our fish model is an adaptive response to a high-fat diet for harvesting more energy from the food. High-throughput sequencing may afford important information for dominant bacteria in the environment, but nondominant bacteria that also have the potential to influence a bacterial community composition in the intestine should not be ignored in such analyses. Sequencing-guided research and function-guided research for identification of bacterial function are both necessary and important. Understanding the role of intestinal microbiota in diet-driven metabolic disorders in different model organisms is a consequential topic not only for intervention of metabolic disorders in animals but also for revealing the as yet incompletely comprehended symbiotic relationship between the host and intestinal microbiota across the tree of life.

## MATERIALS AND METHODS

### Ethics statement.

All experiments were performed under the guidance of the care and use of laboratory animals in China. This research was approved by the Committee on the Ethics of Animal Experiments of East China Normal University (ECNU) (no. F20140101).

### Bacterial screening and identification.

The intestinal contents of six Nile tilapia fed with a high-fat diet (Table S1) for 8 weeks were collected and plated on a medium with soybean oil as the main carbon source (Table S2) for 24 h at 28°C aerobically. Afterwards, 200 colonies were successfully isolated from agar plates and analyzed by amplified rRNA gene restriction analysis (ARDRA; detailed information is provided in the supplemental material). The most abundant bacterium based on ARDRA was selected and designated S1. The nearest neighbors of bacterium S1 were identified by using RDP SEQMATCH, and phylogenetic analysis was performed with MEGA 7.0. Distance was calculated based on the neighbor-joining criterion. The bootstrap confidence values were obtained based on 100 replicates.

10.1128/mSystems.00303-20.2 TABLE S1 Formulation and nutritional composition of experimental diet. Download Table S1, DOCX file, 0.01 MB. Copyright © 2020 Zhang et al. 2020 Zhang et al. This content is distributed under the terms of the Creative Commons Attribution 4.0 International license.

10.1128/mSystems.00303-20.3 TABLE S2 Culture medium for bacterial enrichment. Download Table S2, DOCX file, 0.01 MB. Copyright © 2020 Zhang et al. 2020 Zhang et al. This content is distributed under the terms of the Creative Commons Attribution 4.0 International license.

### Experimental design for fish culturing.

The details of the fish husbandry protocols are provided in the supplemental material. There were two separate feeding trials in this study. In order to verify whether S1 can reach and proliferate in the intestine of the fish, one colony was picked and a spontaneous mutant for rifampin resistance was selected and added to the diet of fish in the first trial. In this trial, fish were randomly distributed into two treatments. In one treatment, fish were fed with the control diet, and in the other treatment, fish were supplemented with 10^9^ CFU of rifampin-resistant S1 g^−1^ of diet every day. This trial lasted for 8 weeks. Each treatment included three tanks, and each tank contained 16 fish. During the experiment, feces were squeezed from live fish from the 4th week to the 8th week. A tryptic soy agar (TSA) medium supplemented with 700 μg ml^−1^ of rifampin was used to culture the feces of fish, and the bacteria with rifampin resistance were quantified (Table 1).

Based on the results of the above-described experiment, we conducted the second feeding trial. Bacterium S1 without rifampin resistance was used in this trial. After acclimatization, all fish were randomly distributed into 12 sterile 99-liter tanks (16 fish per tank). In order to identify the function of bacterium S1 *in vivo*, four treatments (each treatment included triplicate tanks), including CON (fed with basal diet), CONB (fed with basal diet with 10^9^ CFU of S1 g^−1^ of diet added), HF (fed with high-fat diet), and HFB (fed with high-fat diet with 10^9^ CFU of S1 g^−1^ of diet added), were set. The diet components are shown in Table S1. This trial lasted for 8 weeks.

No fish died during the experiment. At the end of the trials, the fish with top 10% and bottom 10% body weights were excluded and a total of 38 fish with relatively similar weights were used for further analysis. Among the 38 fish, 3 individuals from each tank (a total of 9 fish in each treatment) were collected for whole-body lipid content assay. Two individuals from each tank (a total of 6 fish in each treatment) were used for RNA extraction, real-time PCR quantification, and biochemical analysis. Six fish were administered oil by oral gavage to detect lipid absorption. Six fish in each treatment were used for intestinal permeability (FITC-dextran gavage experiment). Six fish in each treatment were collected for Ussing chamber analysis. Five individuals were used for intestinal bacterial genomic DNA extraction.

### Quantitative analyses of bacterium S1 in fish gut.

In order to detect the abundance of bacterium S1 in fish gut, intestinal bacterial DNA was extracted from 5 individuals in each treatment. The PCR fragment which is specific for *Citrobacter* spp. (26) was amplified from the genomic DNA of bacterium S1 and ligated into the vector. The standard curve was made by using diluted linearized plasmid (27). Each assay was performed in triplicate. The cycle threshold of each sample was then compared with the standard curve.

### Body composition and tissue sampling.

After the second trial, 9 fish from each treatment (3 fish from each tank) were dried and milled individually, and the fish powder samples of the 3 fish from the same tank were pooled for the total fat assay (*n* = 3 pooled samples per treatment). Total fat contents of whole fish were determined by chloroform-methanol (CM) methods according to reference 28. Briefly, the fish powder was mixed with CM solution (CHCI_3_- CH_3_OH = 2:1) and stored overnight in a refrigerator at 4°C. After the addition of 1 ml of 0.88% KCl, the mixed solution separated into two layers, and the chloroform layer was removed and dried for analysis. The contents of total fat were calculated according to the ratio of fat to whole fish powder.

Fish were fasted overnight and then euthanized using tricaine methanesulfonate (MS-222, 25 mg liter^−1^). Blood was taken from the tail vein after measuring the body weight. Liver and mesenteric adipose tissue were collected from 6 fish in each treatment and weighed. Intestinal tissue of foregut from each individual was collected for RNA extraction. All tissue samples were frozen in liquid nitrogen and preserved at –80°C until analyzed.

### Quantification of triglyceride and free fatty acids in gut content and serum.

Soybean oil (10 μl g^−1^ of fish body weight) was administered by oral gavage to 6 fish in each treatment according to their body weight after starvation for 12 h. After 90 min, fish were euthanized with MS-222 (25 mg liter^−1^) and blood samples were collected and centrifuged at 3,000 × *g* for 10 min to obtain the serum. The intestinal content of the whole gut of these fish was collected. The concentrations of triglycerides and free fatty acids in the intestinal content and serum were determined by triglyceride assay kit and nonesterified free fatty acid assay kit (Jiancheng, Nanjing, China), respectively. All measurements were performed in triplicates in 96-well plates.

### Intestinal permeability *in vivo* and *in vitro*.

FITC-dextran was used to detect the intestinal permeability *in vivo* according to a previous report (29), and an Ussing chamber was used to detect the intestinal permeability *in vitro* (30). Detailed information can be found in the supplemental material.

### RNA isolation and quantitative real-time PCR.

A sample with 20 mg of intestinal tissue from each fish was collected and homogenized in 1,000 μl of lysis buffer. The total RNA was extracted by using Tri Pure reagent (Aidlab, Beijing, China). The quality and quantity of total RNA were detected by a NanoDrop 2000 spectrophotometer and electrophoresis (Thermo Fisher Scientific, Waltham, MA). RNA with an absorbance ratio, i.e., ratio of optical densities at 260 and 280 nm (OD_260/280_), between 1.9 and 2.2 and OD_260/230_ greater than 2.0 was used for subsequent analysis. cDNA was synthesized by using 1,000 ng of total RNA as the template by utilizing a PrimerScript RT reagent kit (RR047A; TaKaRa, Shiga, Japan) according to the manufacturer’s instructions.

Quantitative real-time PCR (qRT-PCR) was conducted to detect the expression levels of genes related to lipid metabolism with six individuals in each treatment. The primers used for qRT-PCR in this study are presented in Table S3. The qRT-PCR was performed in a CFX96 real-time PCR detection system (Bio-Rad, Hercules, CA). The qPCR mixture contained 10 μl of 2× SYBR qPCR mixture (Aidlab Biotech, Beijing, China), 100 ng of cDNA, 300 nM qPCR primers, and nuclease-free water. The qPCR conditions consisted of 1 cycle at 95°C for 30 s followed by 40 cycles at 95°C for 5 s and an annealing step at 60°C for 20 s. The melting curves of the amplified products were analyzed at the end of the qPCR. Each experiment and the negative controls (no cDNA) were performed in triplicates. Two genes stably expressed in different groups, the β-actin and elongation factor 1 alpha (EF1α) genes, were chosen for qPCR normalization. The relative expression levels were analyzed by 2^−ΔΔ^*^CT^*. Δ*C_T_* = *C*_target_ (*C_T_*_EF1α_ + *C_T_*_β-actin_)/2 (31).

10.1128/mSystems.00303-20.4 TABLE S3 Primers used for qRT-PCR expression analysis. Download Table S3, DOCX file, 0.01 MB. Copyright © 2020 Zhang et al. 2020 Zhang et al. This content is distributed under the terms of the Creative Commons Attribution 4.0 International license.

10.1128/mSystems.00303-20.5 TABLE S4 Diversity index of gut bacteria of Nile tilapia in four groups after 8 weeks of diet intervention. Download Table S4, DOCX file, 0.01 MB. Copyright © 2020 Zhang et al. 2020 Zhang et al. This content is distributed under the terms of the Creative Commons Attribution 4.0 International license.

### Intestinal bacterial composition analyses.

The intestinal content of foregut of five fish was collected for total bacterial community DNA purification by using the Qiagen stool DNA extraction kit (Qiagen, Germany). DNA yield was measured in a NanoDrop 2000 (Thermo Scientific, DE).

The community genomic DNA was used as the template for 16S rRNA gene V3-V4 region amplification (Table S3). Unique eight-base barcodes were added to each primer to distinguish PCR products. The PCR amplification mixture (25 μl) included 0.25 U of Platinum Pfx DNA polymerase (Invitrogen, CA), 2.5 μl of the corresponding 10×Pfx amplification buffer, 0.5 mM MgSO_4_, 0.25 mM deoxynucleoside triphosphate (dNTP) mixture, 1 μM each primer, and 20 ng of extracted DNA. The PCR program began with a 3-min denaturation step at 94°C, followed by 20 cycles of 1 min at 94°C (denaturation), a 1-min annealing step (65°C to 57°C with a 1°C reduction every two cycles, followed by one cycle at 56°C and one cycle at 55°C), and a 1-min elongation step at 72°C; there was a final 6-min extension at 72°C. Thirty nanograms of each purified PCR product was subjected to Illumina-based high-throughput sequencing (Majorbio Bio-Pharm Technology Co., Ltd., Shanghai, China).

Raw fastq files were demultiplexed and quality filtered using QIIME (version 1.17). Reads containing more than two mismatches to the primers or more than one mismatch to the barcode were discarded and reads with lengths of <50 bp were removed. Reads (250 bp) were truncated at any site receiving an average quality score of <20 over a 50-bp sliding window. Operational taxonomic units (OTUs) were clustered with a 97% similarity cutoff using UPARSE (version 7.1; http://drive5.com/uparse/), and chimeric sequences were identified and removed using UCHIME (version 4.1). The phylogenetic affiliation of each 16S rRNA gene sequence was analyzed by RDP Classifier (http://rdp.cme.msu.edu/) against the SILVA database using a confidence threshold of 70%.

Rarefaction curves were created in Mothur to determine whether sequencing depth was sufficient to cover the expected number of OTUs at the level of 97% sequence similarity. Taxonomic richness and diversity estimators were determined for each library in Mothur (32). Principal-coordinate analysis (PCoA), based on unweighted UniFrac distance metric, was used to analyze all OTUs, affording information on microbial community differences among samples.

Thirty-one OTUs were selected for heat map analysis based on (i) the average abundance of these OTUs being higher than 0.05% or (ii) the abundance of these OTUs being significantly different among treatments using Kruskal-Wallis in R package (33).

### Statistical analyses.

Data were expressed as means ± standard errors of the means (SEM), and statistical analyses were performed using GraphPad Prism 6. Normal distribution was tested using the Shapiro-Wilk test. Two-way ANOVA was used to test the effects of bacterium S1 treatment, dietary lipid levels, and their interactions. *Post hoc* Tukey’s test was used to identify significance at a *P* value of <0.05. Statistical results are shown in Table S5. Different letters represent significant difference at a *P* value of <0.05.

10.1128/mSystems.00303-20.6 TABLE S5 Statistical calculation of the values in figures. Download Table S5, DOCX file, 0.02 MB. Copyright © 2020 Zhang et al. 2020 Zhang et al. This content is distributed under the terms of the Creative Commons Attribution 4.0 International license.

### Data availability.

The 16S rRNA gene sequence of bacterium S1 is available in GenBank with accession number MK228843, and the sequences for intestinal microbiota composition analyses are deposited in GenBank with accession number PRJNA506618.

10.1128/mSystems.00303-20.1 TEXT S1 Amplified rRNA gene restriction analysis, bacterium identification, fish culture, mesenteric fat index, intestinal permeability *in vivo* and *in vitro*, and detection of LPS binding protein. Download Text S1, DOCX file, 0.02 MB. Copyright © 2020 Zhang et al. 2020 Zhang et al. This content is distributed under the terms of the Creative Commons Attribution 4.0 International license.
