# Supplementary figures and images for: Citrobacter Species Increase Energy Harvest by Modulating Intestinal Microbiota in Fish: Nondominant Species Play Important Functions
Source: mSystems. 2020 Jun 16;5(3):e00303-20. doi: 10.1128/mSystems.00303-20 (PMC7300360; doi:10.1128/mSystems.00303-20)

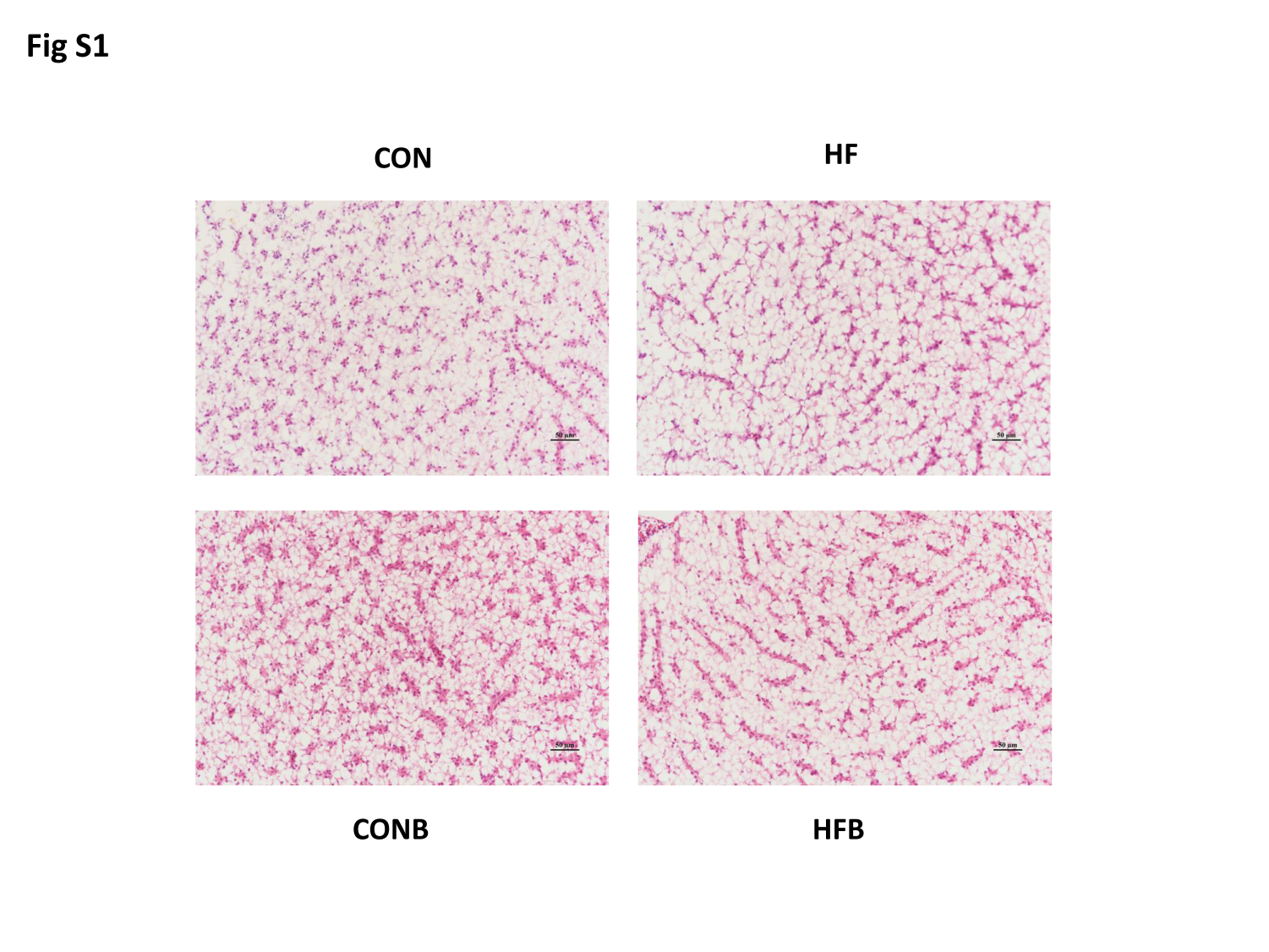

Supplement: FIG S1 [file mSystems.00303-20-sf001.tif]

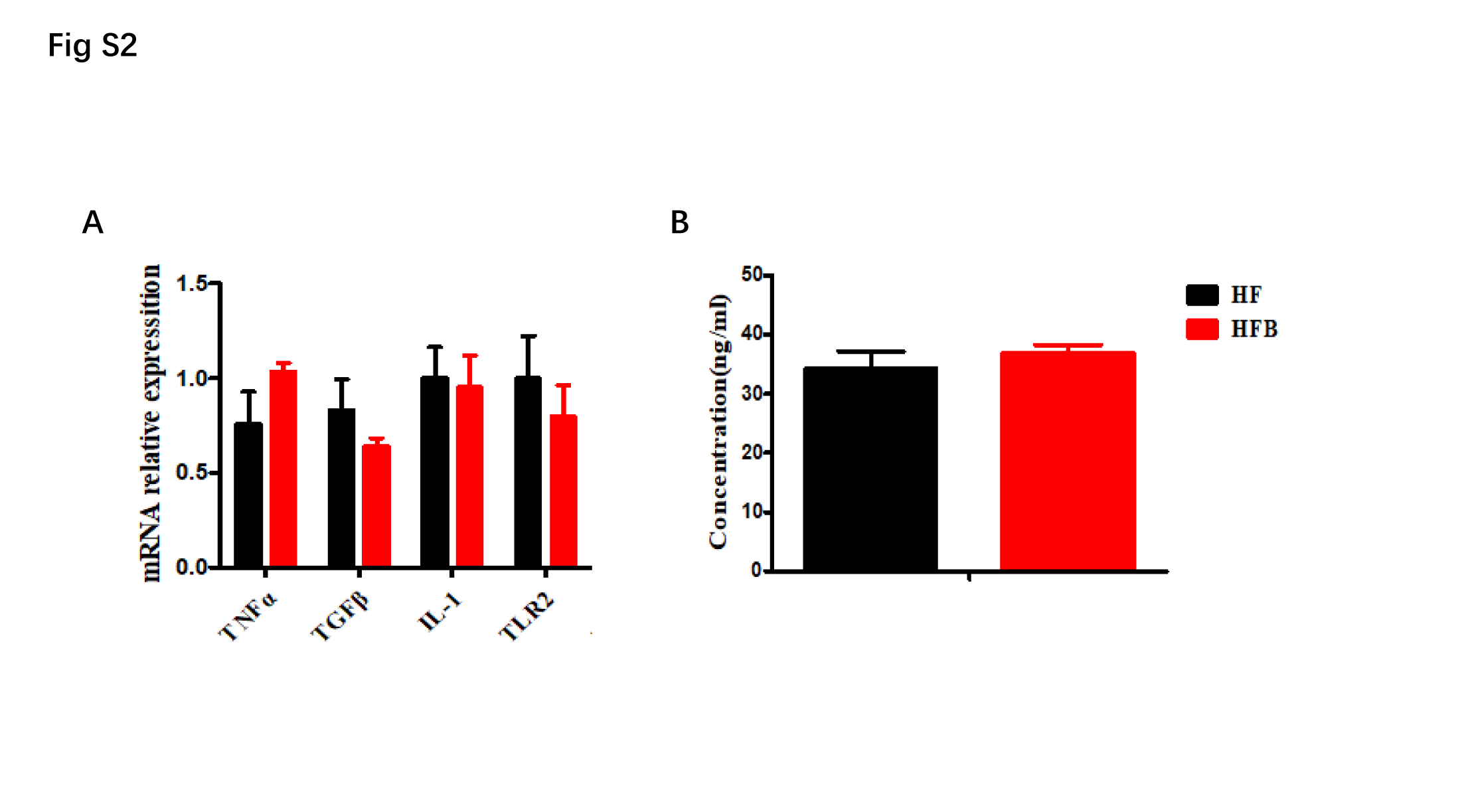

Supplement: FIG S2 [file mSystems.00303-20-sf002.tif]

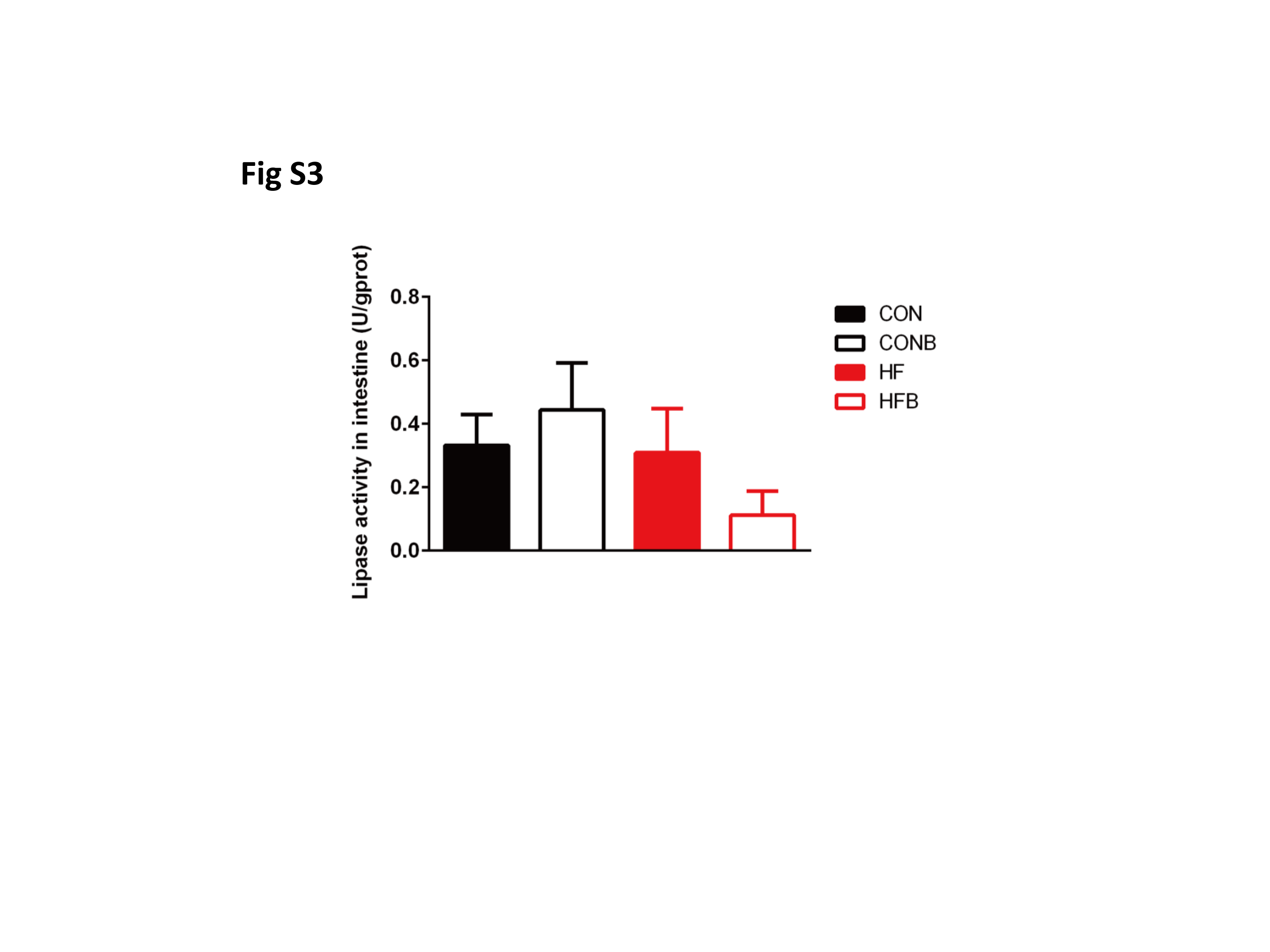

Supplement: FIG S3 [file mSystems.00303-20-sf003.tif]
