# Supplementary material for: Citrobacter Species Increase Energy Harvest by Modulating Intestinal Microbiota in Fish: Nondominant Species Play Important Functions
Source: mSystems. 2020 Jun 16;5(3):e00303-20. doi: 10.1128/mSystems.00303-20 (PMC7300360; doi:10.1128/mSystems.00303-20)
Supplement: TABLE S1 [file mSystems.00303-20-st001.docx]

**Table S1** Formulation and nutritional composition of experimental diet

| Component (g/kg) | Control diet | High fat diet |
| --- | --- | --- |
| Casein | 350 | 350 |
| Gelation | 88 | 88 |
| Corn starch | 150 | 150 |
| Soybean oil | 68 | 164 |
| Mixed vitamin^a^ | 15 | 15 |
| Mixed minerals^b^ | 45 | 45 |
| Carboxy methyl cellulose (CMC) | 30 | 30 |
| Cellulose | 246.75 | 150.75 |
| Choline chloride | 5 | 5 |
| Butylated hydroxytoluene (BHT) | 0.25 | 0.25 |
| [Phagostimulant](http://www.so.com/link?url=http%3A%2F%2Fdict.youdao.com%2Fsearch%3Fq%3Dphagostimulant%26keyfrom%3Dhao360&q=%E8%AF%B1%E9%A3%9F%E5%89%82%E8%8B%B1%E6%96%87&ts=1516716778&t=6f5f21666dab88f9656b0df87592e2f) | 2 | 2 |
| Total (g) | 1000 | 1000 |
| Total fat (%) | 6.8 | 16.4 |
| Total protein (%) | 43.88 | 43.88 |

**^a^** mixed vitamin， (mg or IU/kg)： 500,000 I.U. (international units) Vitamin A， 50,000 I.U. Vitamin D3， 2500 mg Vitamin E， 1000 mg Vitamin K3， 5000 mg Vitamin B1， 5000 mg Vitamin B2，5000 mg Vitamin B6， 5000 μg Vitamin B12， 25,000 mg Inositol， 10,000 mg Pantothenic acid，100,000 mg Cholin， 25,000 mg Niacin， 1000 mg Folic acid， 250 mg Biotin 10,000 mg Vitamin C

**^b^** mixed minerals， (g/kg)： 314.0 g CaCO_3_， 469.3g KH_2_PO_4_， 147.4 g MgSO _4_·7H_2_O， 49.8 g NaCl， 10.9 g Fe(II)gluconate， 3.12 g MnSO_4_ ·H_2_O， 4.67 g ZnSO_4_·7H_2_O， 0.62 g CuSO_4_·5H_2_O，0.16 g KJ， 0.08 g CoCl _2_ ·6H 2 O， 0.06 g NH_4_ molybdate， 0.02 g NaSeO_3_
