# Supplementary material for: Citrobacter Species Increase Energy Harvest by Modulating Intestinal Microbiota in Fish: Nondominant Species Play Important Functions
Source: mSystems. 2020 Jun 16;5(3):e00303-20. doi: 10.1128/mSystems.00303-20 (PMC7300360; doi:10.1128/mSystems.00303-20)
Supplement: TABLE S2 [file mSystems.00303-20-st002.docx]

**Table S2** Culture medium for bacterial enrichment

| Ingredients | Concentration in Media |
| --- | --- |
| NaCl, | 5 g liter ^-1^ |
| K_2_HPO_4_ | 0.3g liter ^-1^ |
| KH_2_PO_4_ | 0.3 g liter ^-1^ |
| MgSO_4_·7H_2_O | 0.1 g liter ^-1^ |
| (NH_4_)_2_SO_4_ | 1 g liter ^-1^ |
| Tryptone | 5 g liter ^-1^ |
| Soy bean oil | 1 ml liter ^-1^ |
