# Supplementary material for: Citrobacter Species Increase Energy Harvest by Modulating Intestinal Microbiota in Fish: Nondominant Species Play Important Functions
Source: mSystems. 2020 Jun 16;5(3):e00303-20. doi: 10.1128/mSystems.00303-20 (PMC7300360; doi:10.1128/mSystems.00303-20)
Supplement: TABLE S3 [file mSystems.00303-20-st003.docx]

**Table S3** Primers used for qRT-PCR expression analysis

| **Gene** |  | **Primer Sequence (5’-3’)** | **Accession number**  **or reference** |
| --- | --- | --- | --- |
| Bacteria | | |  |
| Universal | 27F | GAGAGTTTGATCCTGGCTCAG | (1) |
|  | 1492R | GGTTACCTTGTTACGACTT' |  |
| *Citrobacteria* spp. | F | TTGGCGTCCAGCGCATTCA | (6) |
|  | R | AATTCCAGCCTTCGGCAAACG |  |
| V3-V4 region of bacteria | 338F | 5ʹ-ACTCCTACGGGAGGCAGC-3ʹ | H represents either A, T or C; V represents either G, A or C, W represents A or T. |
|  | 806R | 5ʹ- GGACTACHVGGGTWTCTAAT -3ʹ. |  |
| Nile Tilapia (*Oreochromis niloticus*) | | |  |
| *ef1α* | F | ATCAAGAAGATCGGCTACAACCCT | NM_001279647.1 |
|  | R | ATCCCTTGAACCAGCTCATCTTGT |  |
| *β-actin* | F | AGCCTTCCTTCCTTGGTATGGAAT | XM_003443127.5 |
|  | R | TGTTGGCGTACAGGTCCTTACG |  |
| *mgat1* | F | GCTGTTAAGCGAGAAACGGG | XM_025911812.1 |
|  | R | CTGTGGCCATTTCTTCGGGA |  |
| *mgat2* | F | TTGGACCTCCTTCACTCGCTC | XM_003458980.5 |
|  | R | GGGGTATATCCACGGGGGC |  |
| *dgat2* | F | GCTTGAATTCTGTCACCCTGAAGA | XM_003458972.5 |
|  | R | ACCTGCTTGTAGGCGTCGTTCT |  |
| *apob* | F | TCCCCAGCTACACTGCACAGTT | XM_019348549.2 |
|  | R | CATCGCCTCTTCCTGACATCATC |  |
| *cd36* | F | ATCTTCGAACCATCCATGTCAGTG | XM_003452029.2 |
|  | R | GATATGTGATGCTGGAGGAAGCAA |  |
| *cpt1* | F | TTTCCAGGCCTCCTTACCCA | XM_013268638.3 |
|  | R | TTGTACTGCTCATTGTCCAGCAGA |  |
| *tnfα* | F | CAGAAGCACTAAAGGCGAAGAACA | NM_001279533.1 |
|  | R | TTCTAGATGGATGGCTGCCTTG |  |
| *tgfβ* | F | AAGAGGAGGAGGAATACTTTGCCA | XM_025897821.1 |
|  | R | GAAGCTCATTGAGATGACTTTGGG |  |
| *il-1β* | F | CCTACACCCATCGCTGAGAC | XM_019365844.2 |
|  | R | GGGTAGCGGACAGACATGAG |  |
| *tlr2* | F | GTATCTCAGTGCTCGTCGCTCA | XM_019360109.2 |
|  | R | TTTCATTATCGTCTCCAGTGCG |  |
