# Supplementary material for: Citrobacter Species Increase Energy Harvest by Modulating Intestinal Microbiota in Fish: Nondominant Species Play Important Functions
Source: mSystems. 2020 Jun 16;5(3):e00303-20. doi: 10.1128/mSystems.00303-20 (PMC7300360; doi:10.1128/mSystems.00303-20)
Supplement: TABLE S4 [file mSystems.00303-20-st004.docx]

**Table S4** Diversity index of gut bacteria of Nile tilapia in four groups after 8 weeks of diet intervention.

| **Sample^a^** | **Shannon^b^** | **Simpson** | **ACEs** | **Chao1** |
| --- | --- | --- | --- | --- |
| **CON (n=5)** | 2.99±0.58 | 0.22±0.1 | 567.68±65.19 | 569.24±58.32 |
| **CONB (n=3)** | 3.19±0.83 | 0.13±0.08 | 389.46±45.73* | 395.65±49.85* |
| **HF (n=5)** | 2.37±0.47 | 0.27±0.13 | 523.02±96.24 | 512.64±115.77 |
| **HFB (n=5)** | 3.63±0.79^#^ | 0.08±0.04^#^ | 677.88±92.26^#^ | 696.81±97.26^#^ |

**^a^** CON, control group; CONB, control with bacterium S1; HF, high-fat diet group; HFB, high-fat diet group with bacterium S1.

**^b^** Values are means $\pm$ S.E.M. * Compared with CON, P<0.05. # Compared with HF, P<0.05.
