# Supplementary material for: Citrobacter Species Increase Energy Harvest by Modulating Intestinal Microbiota in Fish: Nondominant Species Play Important Functions
Source: mSystems. 2020 Jun 16;5(3):e00303-20. doi: 10.1128/mSystems.00303-20 (PMC7300360; doi:10.1128/mSystems.00303-20)
Supplement: TABLE S5 [file mSystems.00303-20-st005.docx]

| **Table S5** The statistical calculation of the values in Figures | | | | | | |
| --- | --- | --- | --- | --- | --- | --- |
|  |  | Two-way ANOVA | | | |  |
|  |  |  | F×B | Fat(F) | Bacteria(B) |  |
| Fig.1C  Whole body lipid content |  | *F* | 0.4424 | 16.57 | 21.92 |  |
|  |  | *P* | 0.5185 | 0.0016 | 0.0005 |  |
| Fig.1D  Population level of *Citrobacteria spp.* | | *F* | 19.23 | 309.8 | 19.58 |  |
|  |  | *P* | 0.0005 | ＜0.0001 | 0.0005 |  |
| Fig.3A  Liver lipid content | | *F* | 0.2673 | 46.90 | 0.3784 |  |
|  |  | *P* | 0.6114 | ＜0.0001 | 0.5462 |  |
| Fig.3B  Mesenteric fat index | | *F* | 2.949 | 60.42 | 7.677 |  |
|  |  | *P* | 0.1022 | ＜0.0001 | 0.0122 |  |
| Fig.3D  Adipocyte area | | *F* | 5.538 | 60.54 | 5.632 |  |
|  |  | *P* | 0.0223 | ＜0.0001 | 0.0212 |  |
| Fig.4B  Free fatty acid in intestinal contents | | *F* | 7.814 | 1.091 | 16.64 |  |
|  |  | *P* | 0.0234 | 0.3268 | 0.0035 |  |
| Fig.4C  Triglyceride in serum | | *F* | 0.7166 | 12.49 | 4.515 |  |
|  |  | *P* | 0.4192 | 0.0064 | 0.0626 |  |
| Fig.4D  Free fatty acid in serum | | *F* | 0.4046 | 34.11 | 2.728 |  |
|  |  | *P* | 0.5527 | 0.0021 | 0.1595 |  |
| Fig.4E  ApoB in serum | | *F* | 4.030 | 26.40 | 105.4 |  |
|  |  | *P* | 0.0660 | 0.0002 | ＜0.0001 |  |
| Fig.4F  *mgat2* | | *F* | 4.304 | 14.72 | 25.86 |  |
|  |  | *P* | 0.0602 | 0.0024 | 0.0003 |  |
| Fig.4F  *dgat2* | | *F* | 14.53 | 23.75 | 4.265 |  |
|  |  | *P* | 0.0088 | 0.0028 | 0.0845 |  |
| Fig.4F  *apob* | | *F* | 0.03757 | 6.209 | 28.81 |  |
|  |  | *P* | 0.8499 | 0.0299 | 0.0002 |  |
| Fig.5A  FITC-dextran | | *F* | 4.805 | 10.85 | 14.87 |  |
|  |  | *P* | 0.0435 | 0.0046 | 0.0014 |  |
| Fig.5B  Ω | | *F* | 103.1 | 142.6 | 446.9 |  |
|  |  | *P* | ＜0.0001 | ＜0.0001 | ＜0.0001 |  |
| Table S4  Shannon | | *F* | 3.172 | 6.085 | 0.08495 |  |
|  |  | *P* | 0.0966 | 0.0272 | 0.7750 |  |
| Table S4  Simpson | | *F* | 1.304 | 11.54 | 0.003421 |  |
|  |  | *P* | 0.2727 | 0.0043 | 0.9542 |  |
| Table S4  ACEs | | *F* | 20.05 | 0.09857 | 10.74 |  |
|  |  | *P* | 0.0005 | 0.7582 | 0.0055 |  |
| Table S4  Chao1 | | *F* | 18.85 | 0.01647 | 8.807 |  |
|  |  | *P* | 0.0007 | 0.8997 | 0.0102 |  |
