# Supplementary material for: Citrobacter Species Increase Energy Harvest by Modulating Intestinal Microbiota in Fish: Nondominant Species Play Important Functions
Source: mSystems. 2020 Jun 16;5(3):e00303-20. doi: 10.1128/mSystems.00303-20 (PMC7300360; doi:10.1128/mSystems.00303-20)
Supplement: TEXT S1 [file mSystems.00303-20-s0001.docx]

**Supplementary Methods**

**Amplified ribosomal DNA restriction analysis**

For bacterial identification, the genomic DNA from the bacterial isolates was prepared for 16S rDNA amplification. In brief, bacterial pellets were suspended in 20 µl of Triton-X buffer (0.5% triton-100) and boiled for 10 minutes. The tubes were then centrifuged and the supernatants were used for 16S rRNA gene amplification. PCR products were separated by amplified ribosomal DNA restriction analysis (ARDRA) by digesting with Hinf I and Alu I. The bacterium which was more dominant *in vitro* was selected based on ARDRA profile and sequenced in Majorbio Bio-Pharm Technology Co., Ltd., (Shanghai, China).

**Bacteria identification**

The genomic DNA purified from the bacterium S1 was used for 16S rRNA gene sequencing (Tiangen, Beijing, China). PCR was performed in a 25-μl reaction containing 20ng bacterial genomic DNA, 200 mM (each) deoxynucleoside triphosphates, 1 U of TaKaRa rTaq polymerase (Takara, Dalian), 1×reaction buffer and 0.2 mM of each primer 27F and 1492R (1, 2) (Table S3). A 25 cycles PCR program was performed according to (3). 16S rRNA gene was sequenced in Majorbio Bio-Pharm Technology Co., Ltd., (Shanghai, China).

**Fish culture**

About 300 Nile Tilapia (*Oreochromis niloticus*) were purchased from Shanghai Ocean University (Shanghai, China). Fish were supplied with compressed air via air-stones from air pumps at a 10 h/14 h light/dark cycle and water temperature ranges from 26 to 28 °C, dissolved oxygen was higher than 4.0 mg/L, and pH and total ammonia nitrogen were maintained at 7.5 to 8 and < 0.02 mg/L, respectively. During the two-week acclimatization, fish were hand-fed using a commercial diet (Chengdu, China) containing ≥ 33% protein and ≥ 5% lipid. All fish were hand-fed twice daily between 9:00 and 10:00 h and 17:00 and 18:00 h at 4% of their average body weight per day for 8 weeks. The weight of individual fish was recorded every two weeks and the feed rations were adjusted accordingly. Half of the water was replaced with fresh dechlorinated water every 24 hours.

**Mesenteric fat index**

Mesenteric fat index was calculated by the ratio of mesenteric fat weight to the body weight.

**Intestinal permeability in vivo and in vitro**

FITC-dextran was used to detect the intestinal permeability *in vivo* (4). Fish were starved for 12 hours before the start of the experiment. Five fish were involved in each group and each fish was weighed and an appropriate volume of 50mg ml^-1^ FITC-dextran (4kDa; Sigma-Aldrich, Shanghai, China) dissolved in water was orally gavaged to fish to achieve 50 mg kg^-1^. After 30 minutes, blood samples were collected and centrifuged at 3 000 ×g for 10 minutes to obtain the serum. The serum was further diluted 1 000 times with PBS solution and fluorescence was detected using an F-4500 fluorescence spectrophotometer (HITACHI, Japan) using excitation wavelength of 480nm and emission wavelength of 530nm. The concentration of FITC-dextran in the serum was calculated according to a standard curve generated with known concentrations of pure FITC-dextran.

Ussing chamber was used to detect the intestinal permeability *in vitro* (5). Intact tilapia intestines were cut open and around 0.01cm^2^ of tissue were rinsed in Ringer’s solution (NaCl, 140 mM; NaHCO_3_, 10 mM; KCl,4 mM; NaH_2_PO_4_,2 mM; MgSO_4_,1 mM; CaCl_2_,1 mM; glucose, 5.5; pH 7.8) and then mounted on P2306 clamps. After an equilibration step of 20 minutes, transepithelial electrical resistance (TER) was automatically recorded every 1 min over a ten-minute period. Six individuals were involved in each group.

**Detection of LPS binding protein**

Fish were euthanized with MS-222 (25mg/L) and blood samples were collected and centrifuged at 3 000 ×g for 10 minutes to obtain the serum. Concentration of LPS binding protein in serum was determined by using an ELISA Kit (Jianglaibio, Shanghai, China).

**Lipase activity detection**

Foregut samples (about 30 mg) were homogenized in precooled phosphate buﬀer according to the mass volume ratio of 1/9 to make a 10% homogenate. The sample was centrifuged for 20 min (4℃, 3000 rpm). The supernatant was used to determine the lipase enzyme activities. Lipase activity was measured using a commercially available kit (A054-2-1) purchased from Nanjing Jiancheng Bioengineering Institute (Nanjing,China).

**References**

1. Di Cello F, Bevivino A, Chiarini L, Fani R, Paffetti D, Tabacchioni S, Dalmastri C.1997. Biodiversity of a Burkholderia cepacia population isolated from the maize rhizosphere at different plant growth stages. Appl Environ Microbiol 63:4485-4493.

2. Hayashi H, Sakamoto M, Benno Y. 2002. Phylogenetic analysis of the human gut microbiota using 16S rDNA clone libraries and strictly anaerobic culture-based methods. Microbiol Immunol 46 :535-548.

3. Eckburg PB, Bik EM, Bernstein CN, Purdom E, Dethlefsen L, Sargent M, Gill SR, Nelson KE, Relman DA. 2005. Diversity of the human intestinal microbial flora. Science. 308:1635-1638.

4. Dodd D, Spitzer MH, Van Treuren W, Merrill BD, Hryckowian AJ, Higginbottom SK, Le A, Cowan TM, Nolan GP, Fischbach MA, Sonnenburg JL*.* 2017. A gut bacterial pathway metabolizes aromatic amino acids into nine circulating metabolites. Nature 551:648-652.

5. Westerhout J, Wortelboer H, Verhoeckx K. 2015. Ussing Chamber. p 263-273. *In* Verhoeckx K, Cotter P, Lopez-Exposito I, Kleiveland C, Lea T, Mackie A, Requena T, Swiatecka D, Wichers H. (ed), The Impact of Food Bioactives on Health: in vitro and ex vivo models. Springer, Cham (CH).

6. Kaclíková E, Krascsenicsová K, Pangallo D, Kuchta T. 2005. Detection and quantification of *Citrobacter freundii* and *C. braakii* by 5′-nuclease polymerase chain reaction. Curr Microbiol 51:229-232.
